# Identification of ochratoxin-*N*-acetyl-L-cysteine as a new ochratoxin A metabolite and potential biomarker in human urine

**DOI:** 10.1007/s12550-019-00360-0

**Published:** 2019-05-10

**Authors:** Franziska Sueck, Jonas Specht, Benedikt Cramer, Hans-Ulrich Humpf

**Affiliations:** Institute of Food Chemistry, Westfälische Wilhelms-Universität Münster, Corrensstr. 45, 48149 Münster, Germany

**Keywords:** Ochratoxin A, Glutathione, *N*-acetyl-L-cysteine, Metabolite, Biomarker, Human urine

## Abstract

Ochratoxin A (OTA) is a nephrotoxic mycotoxin with nephrocarcinogenic potential found in a broad spectrum of food commodities. The mode of action of this compound, as well as its metabolism, is still not fully understood. To determine whether the conjugation of OTA with glutathione plays an important role in human OTA metabolism, an ochratoxin-glutathione conjugate (OTB-GSH), as well as the corresponding urinary metabolite ochratoxin-*N*-acetyl-l-cysteine (OTB-NAC), were synthesized and their structures confirmed by NMR spectroscopy. By means of synthesized stable isotope-labeled *d*_*5*_-OTB-GSH and *d*_*5*_-OTB-NAC references, a sensitive HPLC-MS/MS method has been developed and applied for the screening of human urine samples. OTB-NAC could be detected in 11 of the analyzed 18 urine samples and was quantified in 5 urine samples in the range between 0.023 and 0.176 ng mg^−1^ creatinine. OTB-GSH has not been detected in the urine samples. In OTB-NAC positive samples, this metabolite contributed to a comparable concentration range to the total OTA excretion as the parent compound. This is the first direct analysis of an OTA phase 2 metabolite in humans.

**Electronic supplementary material:**

The online version of this article (10.1007/s12550-019-00360-0) contains supplementary material, which is available to authorized users.

## Introduction

Ochratoxin A (OTA) is one of the most widespread mycotoxins produced by fungi of the *Aspergillus* and *Penicillium* genera. OTA exhibits a broad spectrum of toxic effects, with nephrotoxicity and carcinogenicity in animal studies being most prominent (Ringot et al. 2006; Suzuki et al. 1975). The toxin has been classified as possibly carcinogenic to humans by the IARC (group 2B) due to an observed increase of the incidence of hepatocellular tumors and renal cell adenomas in rodents (Ostry et al. 2017; Kőszegi and Poór 2016; EFSA 2006; IARC 1993; Kanisawa and Suzuki 1978; Suzuki et al. 1975). Although several toxic effects of OTA have been well described, the mechanisms of OTA-induced toxicity are still not fully understood (Ringot et al. 2006). For example, the phenylalanine moiety of OTA has been discussed as the responsible substructure for competitive inhibition of enzymes needed for protein biosynthesis (Rottkord et al. 2017; Cramer et al. 2010; McMasters and Vedani 1999; Creppy et al. 1984). The generation of reactive oxygen species (ROS) has furthermore been considered responsible for OTA-induced toxicity and carcinogenicity (Tao et al. 2018). Oxidative metabolism of OTA resulting in OTA metabolites such as reactive quinone structures has also been observed (Dai et al. 2002; Calcutt et al. 2001; Gillman et al. 1999). Accordingly, chemical reaction products of these compounds such as glutathione (GSH) conjugates have recently been detected in cell culture and in rodents (Hadjeba-Medjdoub et al. 2012; Tozlovanu et al. 2012; Jennings-Gee et al. 2010; Dai et al. 2002; El Adlouni et al. 2000; Pfohl-Leszkowicz et al. 1993). A further explanation for the formation of glutathione conjugates of OTA could be the direct transformation with glutathione *S*-transferase (GST). It is known that GST can eliminate halogens from aromatic compounds and substitute with glutathione, like for example sulfobromophthalein sodium (Marquardt et al. 2013; Combes and Stakelum 1961). If this is also the case for OTA, the formation of reactive quinone structures might not be required.

**Fig. 1:**
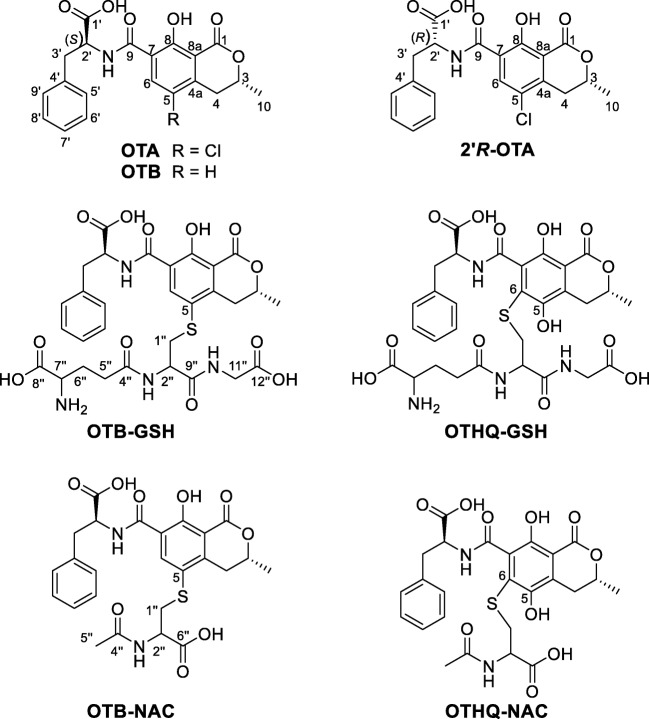
Chemical structures of ochratoxin A (OTA), its diastereomer 2′*R*-ochratoxin A (2′*R*-OTA), its dehalogenated form ochratoxin B (OTB), and its glutathione conjugate (OTB-GSH) and *N*-acetyl-l-cysteine conjugate (OTB-NAC) as well as the ochratoxin hydroquinone conjugates with GSH (OTHQ-GSH) and NAC (OTHQ-NAC) (GSH or NAC-conjugates of OTA are named OTB-GSH and OTB-NAC as the backbone moiety represents the dehalogenated OTB)

El Adlouni et al. (2000) have studied the role of OTA metabolites for potential DNA adduct formation by ^32^P-postlabeling assays. To this end, kidney microsomes from rabbits and human bronchial epithelial cells have been used to demonstrate that after incubation with phenobarbital and the consequently increasing activity of cytochrome P450 and GST rise of signals, corresponding to DNA adduct formation, could be detected. A simultaneous incubation with phenobarbital and ethacrynic acid, which is an inhibitor for GST, showed a significant decrease (El Adlouni et al. 2000). Dai et al. (2002) could detect the OTA glutathione metabolite ochratoxin hydroquinone-glutathione (OTHQ-GSH, Fig. 1) after incubation of OTA with rat microsomes in a NADPH regenerating system without and with additional GSH. Ten years later, Tozlovanu et al. (2012) were able to detect both OTA glutathione conjugates, OTHQ-GSH and ochratoxin-glutathione (OTB-GSH, Fig. 1), in rat liver and kidney after feeding the rats with OTA. The results suggest that OTA undergoes metabolism to generate electrophiles that can react with GSH in rat liver and kidney (Tozlovanu et al. 2012).

The formation of these GSH conjugates has shown the importance of OTA metabolites for the comprehensive understanding of the mode of action of OTA. In addition to the formation of OTA-GSH conjugates, the occurrence of *N*-acetyl-l-cysteine (NAC) conjugates (OTHQ-NAC and OTB-NAC; both Fig. 1) as their most probable renal excretion products is conceivable (Ringot et al. 2006; Nelson 1992). These compounds would be of high importance as potential biomarkers, as enzymatic cleavage of GSH in the kidney via γ-glutamyltransferase and dipeptidase leads to the corresponding cysteine conjugate, which is *N*-acetylated by *N*-acetyltransferase to yield OTB-NAC (Nelson 1992).

Besides GSH conjugates, other phase 2 metabolites of OTA such as glucuronides have only been detected by indirect methods. Their occurrence in human urine has been assumed based on an increasing OTA concentration in urine after enzymatic incubation with β-glucuronidase/arylsulfatase (Muñoz et al. 2017; Duarte et al. 2011). The formation of three OTA-glucuronides after incubation of OTA with rat microsomes has also been proposed based on mass spectrometric data (Han et al. 2013)

Whether the activation and metabolism of OTA with GSH are also of relevance in humans is not known so far. Therefore, to study whether OTB-GSH and OTB-NAC are also human metabolites of OTA, we chemically synthesized both compounds as well as their stable isotope-labeled analogues. Furthermore, a sensitive stable isotope dilution based HPLC-MS/MS method for the analysis of these OTA metabolites in human urine was developed.

## Material and methods

### Materials

Phosphate buffered saline (0.05 mol L^−1^, PBS) was prepared by dissolving 0.19 g KH_2_PO_4_ (1.4 mmol) and 1.65 g Na_2_HPO_4_ (11.6 mmol) in 0.25 L water. The pH was adjusted to pH 10 using NaOH solution (1 mol/L).

Ochratoxin A (OTA) was produced in house according to Sueck et al. (2018b). The isomerization of OTA to 2′*R*-ochratoxin A (2′*R*-OTA) was achieved by heating 20 mg of OTA at 200 °C followed by separation of OTA and 2′*R*-OTA via preparative HPLC-UV, which yielded 2′*R*-OTA in purity of > 99% (Cramer et al. 2008). The stable isotope-labeled standards *d*_*5*_-ochratoxin A (*d*_*5*_-OTA) and *d*_*5*_-2′*R*-ochratoxin A (*d*_*5*_-2′*R*-OTA) were obtained by growing *Penicillium verrucosum* on *d*_*5*_-l-phenylalanine-enriched medium (Cramer et al. 2008). Isotopic purity of both components was > 95%. Chemical structures of the four compounds OTA, 2′*R*-OTA, *d*_*5*_-OTA, and *d*_*5*_-2′*R*-OTA were confirmed by HRMS and NMR spectroscopy and were in agreement with literature data. For each analyte, stock solutions of 10 μg/mL in acetonitrile were prepared and stored at − 20 °C.

### Synthesis and purification of ochratoxin A and *d*_*5*_-ochratoxin A conjugates with GSH and NAC

Coupling of OTA with glutathione (GSH) and *N*-acetyl-l-cysteine (NAC) via photoreaction was carried out according to the method of Hadjeba-Medjdoub et al. (2012). To this end, for the OTB-GSH synthesis, four 15-mL screw capped glass tubes made of Duran glass were loaded each with 10 mg OTA (24.7 μmol), 114 mg GSH (371 μmol), and 10 mL PBS. For the OTB-NAC synthesis, another four 15-mL screw capped glass tubes made of Duran glass were loaded each with 10 mg OTA (24.7 μmol), 60 mg NAC (371 μmol), and 10 mL PBS. Adjusting the pH of the PBS to pH 10 resulted in an about ten times higher yield compared with the commonly used pH 7.4 (data not shown). Glass tubes were placed in a photochemical reactor (Rayonet PRP-200, Southern New England Ultraviolet Company, Brandford, USA) containing RMR-3500 lamps of 12-in. length and irradiated at a wavelength of 350 nm. Irradiation times of 20 min for the preparation of OTB-GSH and of 30 min for OTB-NAC were applied.

For purification of OTB-NAC or OTB-GSH, a Strata C 18-E (10 g, 55 μm, 70 Å, Phenomenex, Aschaffenburg, Germany) SPE column was equilibrated with 20 mL methanol followed by 20 mL water/formic acid (100/0.1, *v*/*v*) and loaded with the respective combined reaction batch. Subsequently, the column was washed with 40 mL of a mixture of water/methanol/formic acid (90/10/0.1, *v*/*v*/*v*) and the reaction product eluted with 30 mL water/methanol/formic acid (40/60/0.1, *v*/*v*/*v*). The solvents were removed via rotary evaporation at 40 °C to obtain the crude reaction products.

The second purification step of OTB-GSH was performed on a Varian ProStar preparative HPLC system (Agilent Technologies) with UV detection at 330 nm. To that end, the crude product obtained from the first purification step was dissolved in 5 mL water/methanol/formic acid (70/30/0.1, *v*/*v*/*v*) and separated at room temperature on a Nucleodur C18 Gravity SB column (250 × 10 mm, 5 μm, Macherey-Nagel, Düren, Germany) equipped with a 4 × 3 mm C-18 guard column (Phenomenex). The following binary linear gradient of methanol containing 0.1% formic acid (A) and water containing 0.1% formic acid (B) was applied at a flow rate of 2 mL min^−1^: 0 min, 30% A, 13 min 100% A, 16 min 100% A, 16.1 min 30% A, 22 min 30% A.

For OTB-NAC, a different cleanup step was applied because of insufficient separation of OTB and OTB-NAC on the column of the preparative reversed phase HPLC-UV system. Crude OTB-NAC obtained after the first purification step was dissolved in toluene/*tert*-butyl methyl ether/formic acid (70/25/5, *v*/*v*/*v*) and purified by elution with the same solvent mixture from a 10 cm × 1 cm silica column (0.040–0.063 mm silica gel 60, Merck, Darmstadt, Germany). Fractions of 5 mL were collected and analyzed by HPLC-DAD for the presence of OTB-NAC. The HPLC-DAD system was a Nexera XR HPLC system consisting of a LC-20 AD XR pump, a DGU 20A5R degasser, a SIL-20 AC XR autosampler, a CTO-10 AS VP column compartment, and a SPR-M30A DAD detector, operated by a CBM-20A controller (Shimadzu, Duisburg, Germany). UV/vis spectra were recorded in a wavelength range of 200–450 nm. For data acquisition and evaluation, Xcalibur 2.0.7 SP1 software (Thermo Fisher Scientific, Bremen, Germany) was used. Chromatographic separation was achieved on a Reprosil-Pur C18 AQ column (150 × 2 mm, 3 μm; Dr. Maisch GmbH, Ammerbruch-Entringen, Germany) with a 5 × 2 mm guard column of the same material. The following linear binary gradient of methanol (A) and water (B) both containing 0.1% formic acid was applied at a flow rate of 0.2 mL min^−1^: 0 min 50% A, 3 min 50% A, 14 min 100% A, 24 min 100% A, 24.1 min 50% A, 30 min 50% A. The column temperature was set to 40 °C. All fractions of OTB-NAC were combined, and the solvent was removed by rotary evaporation at 40 °C.

For purity assessment of OTB-GSH and OTB-NAC, the same HPLC-UV method as described for the analysis of the silica column fractions was used. Before gravimetric measurements on a microbalance, all materials were dried for at least 12 h at room temperature under a vacuum of < 0.1 mbar (Schlenk line). In this way, we obtained 1.20 mg OTB-GSH and 1.76 mg OTB-NAC with a purity of > 99% according to HPLC-UV (220 nm), HPLC-ELSD, and ^1^H-NMR analysis.

For the synthesis of the isotope-labeled standards *d*_*5*_-OTB-GSH and *d*_*5*_-OTB-NAC, the reactions were performed in the same way but on a smaller scale with 2.5 mg *d*_*5*_-OTA as starting material. In total, 5 μg of *d*_*5*_-OTB-NAC and 75 μg *d*_*5*_-OTB-GSH with purities of > 92% and > 93%, respectively, were obtained. The concentrations of stock solutions of the isotope-labeled standards were photometrically determined based on the extinction coefficients of unlabeled OTB-GSH and OTB-NAC, determined as described below.

### Photometric determination of the extinction coefficients of OTB-GSH and OTB-NAC

Determination of the extinction coefficients was done by dissolving 1.20 mg OTB-GSH and 1.76 mg OTB-NAC individually in 2.00 mL methanol/water/formic acid (70/30/0.1, *v*/*v*/*v*). Aliquots of the solutions were diluted with the same solvent mixture to concentrations of approx. 40µg mL^-1^ and UV-Spectra were recorded on a Jasco V-750 UV-photometer (Jasco Deutschland GmbH, Groß-Umstadt, Germany). The extinction coefficients were calculated for the absorption maxima observed at a wavelength of 331 nm. For OTB-GSH, extinction coefficients of 3181 L mol^−1^ cm^−1^ and, for OTB-NAC, of 3575 L mol^−1^ cm^−1^ were determined, based on the following equation:

$$ \upvarepsilon =\frac{\mathrm{A}}{\mathrm{c}\times \mathrm{d}} $$

The extinction coefficient (*ε*) is defined as the absorption (*A*) divided into a mathematical product of the concentration (mol L^−1^) and the length of the cuvette (1 cm). For the determination of the amount of *d*_*5*_-OTB-GSH and *d*_*5*_-OTB-NAC, the purified compounds were each solved in methanol/water/formic acid (70/30/0.1, *v*/*v*/*v*) and the photometric determination was performed as described above at a wavelength of 331 nm.

### Exact mass determination

The exact mass and the product ions of OTB-GSH, *d*_*5*_-OTB-GSH, OTB-NAC, and *d*_*5*_-OTB-NAC were recorded on an LTQ Orbitrap XL mass spectrometer (Thermo Fisher Scientific) operated in positive heated electrospray mode by direct infusion of solutions containing 5 μg mL^−1^ of the respective compound in methanol/water/formic acid (70/30/0.1, *v*/*v*/*v*). Sheath gas flow of the mass spectrometer was set to 5 arbitrary units, and the capillary temperature was adjusted to 275 °C. The source voltage was 4 kV, capillary voltage 21 V, and tube lens 112 V. For data acquisition and evaluation, Xcalibur 2.0.7 SP1 software (Thermo Fisher Scientific) was used.

Product ion spectra were recorded from the protonated molecular ion, isolated with an isolation width of 1.0 *m*/*z.* Fragmentation was done in the HCD cell of the mass spectrometer with a relative collision energy of 35%, and the following mass spectra were acquired with a mass resolution of 60,000:

- OTB-GSH (product of *m*/*z* 675.10): fragments *m*/*z* 600.1642 (35% rel. intensity), *m*/*z* 447.0853 (50% rel. intensity) and *m*/*z* 296.0586 (100% rel. intensity)
- *d*_5_-OTB-GSH (product of *m*/*z* 680.10): fragments *m*/*z* 605.1959 (15% rel. intensity), *m*/*z* 447.0855 (55% rel. intensity) and *m*/*z* 296.0587 (100% rel. intensity)
- OTB-NAC (product of *m*/*z* 531.10): fragments *m*/*z* 384.0747 (100% rel. intensity), *m*/*z* 366.0641 (25% rel. intensity), *m*/*z* 237.0216 (35% rel. intensity)
- *d*_*5*_-OTB-NAC (product of *m*/*z* 536.10): fragments *m*/*z* 384.0743 (100% rel. intensity), *m*/*z* 366.0637 (25% rel. intensity), *m*/*z* 237.0213 (35% rel. intensity)

Heated electrospray ionization was applied with a sheath gas flow of 5 arbitrary units. The capillary temperature was set to 275 °C. The source voltage was 4 kV, capillary voltage 21 V, and tube lens 112 V. For data acquisition and evaluation, Xcalibur 2.0.7 SP1 software (Thermo Fisher Scientific) was used.

### NMR spectroscopy

NMR experiments were carried out on an Agilent DD2 600 MHz spectrometer (Agilent Technologies) with samples dissolved in a mixture of *d*_*4*_-methanol/D_2_O/*d*_*2*_-formic acid (70/30/0.1, *v*/*v*/*v*) due to the poor solubility of the OTA conjugates. Chemical shifts *δ* are reported as parts per million (ppm) in relation to tetramethylsilane. Data processing was done using MestReNova software v. 10 (Mestrelab Research, Santiago de Compostela, Spain).

### Urine sample preparation and analysis

For the analysis of OTA metabolites, spot urine samples provided by 18 volunteers collected within a previous project by Sueck et al. (2018a) were available for this study. All participants were informed about the aim of the study and gave written consent. To 20 mL of each urine sample, 20 μL formic acid was added and the samples were stored at − 20 °C until analysis. To 5 mL urine, 25 μL of the internal standard solution containing *d*_*5*_-OTA, *d*_*5*_-2′*R*-OTA, *d*_*5*_-OTB-GSH, and *d*_*5*_-OTB-NAC (60 ng mL^−1^, each) was added. A Bond Elut Plexa column (200 mg, 6 mL, Agilent Technologies) was washed with 15 mL MeOH and conditioned with 15 mL water/formic acid (100/0.1, *v*/*v*) before 5 mL of urine was passed through the column. Subsequently, the column was washed with 10 mL water/formic acid (100/0.1, *v*/*v*) and 10 mL methanol/water/formic acid (40/60/0.1, *v*/*v*/*v*). OTA, OTB-NAC, and OTB-GSH were eluted with 4 mL acetonitrile. Subsequently, the eluate was evaporated to dryness at 45 °C in a gentle stream of nitrogen and reconstituted in 100 μL methanol/water/formic acid (10/90/0.1, *v*/*v*/*v*). Second sample preparation and analysis was done for all samples that contained OTB-NAC > LOD.

A seven-point calibration curve of OTA, 2′*R*-OTA, OTB-GSH, and OTB-NAC in the range between 0.01–20 ng mL^−1^ and their corresponding stable isotope-labeled standards with 15 ng mL^−1^ each was used for quantitation. The limit for quantification (LOQ) was defined by the lowest calibration point with a signal-to-noise (S/N) ratio > 10.

#### Urine HPLC-MS/MS analysis

Urine samples were analyzed on an Agilent 1260 Infinity LC system coupled to a QTRAP 6500 mass spectrometer (Sciex, Darmstadt, Germany). For chromatographic separation, a Reprosil-Pur C18 AQ column (150 × 2 mm, 3 μm, Dr. Maisch) with a 5 × 2-mm guard column of the same material was used at a temperature of 40 °C. The following linear binary gradient of methanol with 1% formic acid (A) and water with 1% formic acid (B) was applied at a flow rate of 0.2 mL min^−1^: 0 min 10% A, 2 min 10% A, 5 min 50% A, 14 min 100% A, 18.0 min 100% A, 18.1 min 10% A, 24 min 10% A.

The mass spectrometer was operated in the positive and negative electrospray ionization mode with + 5.5 kV and − 4.5 kV ionization voltage, respectively. The source temperature was set to 450 °C; 30 psi curtain gas, 35 psi nebulizer gas (gas 1), and 45 psi desolvation gas (gas 2) were applied. A volume of 40 μL was injected. The analytes were detected in the selected reaction monitoring (SRM) mode (Table 1). The entrance potential and collision cell exit potential were set to − 8 V and − 15 V in negative and to 10 V and 21 V in positive mode, respectively. Data acquisition and quantification were done with Analyst 1.6.2 software (Sciex). For additional confirmation of the identity of OTB-NAC detected in urine, one of the samples found positive for OTB-NAC was reanalyzed using the same gradient and HPLC-MS/MS method as described above, but a Nucleodur Phenyl-Hexyl column (100 × 2.0 cm; 3 μm, Macherey-Nagel) was used for chromatographic separation. Also on this column, identical retention times of the three SRM transitions of the OTB-NAC signal in the reference standard and in the urine sample were observed (data not shown).

**Table 1:** Optimized SRM parameters applied for the analysis using the QTRAP 6500 mass spectrometer

Mode	Analyte		Q1 (*m*/*z*)	Q3 (*m*/*z*)	Dwell time (ms)	Declustering potential (V)	Collision energy potential (V)
Negative	OTB-NAC	Quantifier	528.9	400.1	30	− 30	− 25
		Qualifier 1	528.9	312.0	30	− 30	− 40
		Qualifier 2	528.9	208.1	30	− 30	− 40
	*d*_*5*_-OTB-NAC	Quantifier	533.9	405.1	30	− 30	− 25
		Qualifier	533.9	317.0	30	− 30	− 40
Positive	OTA/ 2′*R*-OTA	Quantifier	404.1	239.0	30	70	31
		Qualifier 1	404.1	221.0	30	70	47
		Qualifier 2	404.1	102.0	30	70	88
	*d*_*5*_–OTA/ *d*_*5*_–2′*R*–d_5_–OTA/ d_5_–2'R-OTA	Quantifier	409.1	239.0	30	35	31
		Qualifier	409.1	102.0	30	35	88
	OTB-GSH	Quantifier	675.2	277.9	30	70	60
		Qualifier 1	675.2	296.1	30	70	45
		Qualifier 2	675.2	600.1	30	70	35
	*d*_*5*_-OTB-GSH	Quantifier	680.2	296.1	30	70	45
		Qualifier	680.2	277.9	30	70	60

#### Creatinine measurements

The creatinine concentrations of the urine samples were determined by a modified *Jaffee* method as described in Sueck et al. (2018a).

#### Urine statistical analysis

Data are presented as mean ± standard error mean (SEM).

## Results and discussion

Two OTA derivatives, OTB-GSH and OTB-NAC, were synthesized by the photoreaction of OTA with GSH or NAC according to the method of Hadjeba-Medjdoub et al. (2012) with slight modifications. Compared with the original method, the pH of the buffer was adjusted to pH 10. After irradiation with UV light at 350 nm, the derivatives were isolated from the reaction mixture by solid phase extraction (SPE), followed by preparative RP-HPLC or additional silica column chromatography. The chemical structures of the obtained reaction products were elucidated by means of high-resolution mass spectrometry (HRMS) and UV- and NMR spectroscopy. HRMS analysis confirmed the expected sum formulas of C_30_H_34_N_4_O_12_S for OTB-GSH and C_25_H_26_N_2_O_9_S for OTB-NAC reported in literature (HRMS data can be found in the Supplementary Information). The UV spectra with maxima at 331 nm for OTB-GSH and OTB-NAC were also in agreement with literature data (Tozlovanu et al. 2012).

For structure elucidation by NMR spectroscopy, one-dimensional (^1^H, ^13^C) and two-dimensional experiments (^1^H,^1^H-COSY, ^1^H,^13^C-HMBC, ^1^H,^13^C-HSQC) were carried out. The proton shifts in Table 2 and the carbon shifts in Table 3 of the synthesized metabolites are compared with OTA. Based on MS, UV, and NMR data, the two synthesized compounds could be unequivocally identified as OTB-GSH and OTB-NAC.

**Table 2:** Chemical shift of the ^1^H-NMR signals of OTB-GSH and OTB-NAC in comparison to OTA (see Fig. 1 for structure and numbering) (NMR measurements were performed in *d*_*4*_-methanol/D_2_O/*d*_*2*_-formic acid (70/30/0.1, *v*/*v*/*v*), literature data of OTB-NAC by Tozlovanu et al. 2012 were obtained in *d*_6_-DMSO)

No. H	OTB-GSH	OTB-NAC	OTB-NAC (Tozlovanu et al. 2012)	OTA
	δ ^1^H (ppm)	δ ^1^H (ppm)	δ ^1^H (ppm)	δ ^1^H (ppm)
3	4.79	4.80	4.39	4.85
4.1/4.2	2.98 ו 3.55	2.92 ו 3.52	2.55 ו 3.35	2.91 ו 3.35
6	8.35	8.40	8.06	8.18
10	1.56	1.55	1.34	1.56
2′	4.85	4.93	4.43	4.92
3'.1/3'.2	3.18 ו 3.31	3.20 ו 3.34	3.08 3.12	3.20 ו 3.32
5′–9′	7.31–7.18	7.31–7.22	7.23–7.19	7.32–7.23
1''.1/1''.2	3.03 ו 3.32	3.14 ו 3.38	2.81 ו 3.06	−
2″	4.27	4.39	3.91	−
5″	2.55	1.96	1.75	−
6″	2.23–2.08	−	−	−
7″	3.69	−	−	−
11″	3.81	−	−	−

**Table 3:** Chemical shifts of the ^13^C-NMR signals of OTB-GSH and OTB-NAC in comparison with OTA (see Fig. 1 for structure and numbering) (NMR measurements were performed in *d*_*4*_*-*methanol/D_2_O/*d*_*2*_-formic acid (70/30/0.1, *v*/*v*/*v*))

No. C	OTB-GSH	OTB-NAC	OTA
	δ ^13^C (ppm)	δ ^13^C (ppm)	δ ^13^C (ppm)
1	171.6	171.6	171.1
3	77.8	77.8	77.6
4	33.9	34.0	32.9
4a	149.4	149.0	143.5
5	123.2	124.1	124.2
6	144.8	144.3	138.6
7	120.5	120.5	120.9
8	161.4	161.2	159.7
8a	111.3	111.2	111.6
9	165.3	165.1	164.7
10	20.9	20.8	20.7
1′	175.9	174.7	174.7
2′	56.5	55.6	55.5
3′	38.4	38.2	38.1
4′	138.0	137.5	137.3
5′ and 9′	130.5	130.4	130.4
6′ and 8′	129.5	129.5	129.5
7′	127.9	128.1	128.1
1″	37.4	37.8	−
2″	53.8	53.5	−
4″	175.3	173.6	−
5″	32.7	22.5	−
6″	27.5	164.9	−
7″	55.3	−	−
8″	174.1	−	−
9″	172.7	−	−
11″	42.5	−	−
12″	173.9	−	−

The ^1^H spectra of OTB-GSH and OTB-NAC showed in comparison with OTA two characteristic shifts of signals for the OTB backbone. At position 4, the signal of one of the two protons is deshielded by *δ* 0.2 ppm. This could be explained by a possible interaction of the proton with the glutathione and *N*-acetyl-l-cysteine residues resulting in an electron withdrawing effect. A further difference of ^1^H spectra of OTB-NAC and OTB-GSH compared with OTA was a deshielding of the proton in position 6 by about 0.2 ppm. This could be explained by the missing chlorine atom as well as the electron-withdrawing effects of the thioether group. In literature, ^1^H-NMR, as well as ^1^H,^1^H-COSY, data were available for OTB-NAC (Tozlovanu et al. 2012). However, a direct comparison of these data is not possible because different solvents had been used in the study of Tozlovanu et al. (2012). Here, in order to increase the solubility of OTB-NAC and OTB-GSH, a mixture of *d*_*4*_-methanol and D_2_O with 0.1% *d*_*2*_-formic acid was used for NMR spectroscopy. In this way, we were able to record ^13^C spectra but cannot directly compare the ^1^H-NMR shifts with those from literature that were recorded in *d*_6_-DMSO.

The ^13^C spectra of OTB-GSH and OTB-NAC recorded downfield chemical shifts of C-6 and C-4a in comparison with OTA (Table 3). Compared with chlorine bound to C-5, the coupling of GSH or NAC (-S-C_a_H_b_O_c_N_d_) generates a stronger deshielding of the *ortho* positions of the aromatic system resulting in higher chemical shifts of the two carbon atoms at positions 4a and 6. The chemical shift at the direct binding site (C-5) and the carbons in meta (C-7 and 8a) and para-position (C-8) to the conjugation were only marginally influenced (Hesse et al. 2005). The position at the OTB backbone, that binds GSH and NAC, respectively, was unambiguously identified by ^1^H,^13^C-HMBC experiments. There, the ^3^*J*_C,H_ coupling for both metabolites of the OTB carbon C-5 with the two protons of GSH and NAC H-1″.1 and H-1″.2 was visible and is shown in the Supplementary Information.

Following structure elucidation and purity determination, stock solutions of OTB-NAC and OTB-GSH were prepared and used for determination of the molar extinction coefficients. Coefficients in methanol/water/formic acid (70/30/0.1, *v*/*v*/*v*) were 3181 L mol^−1^ cm^−1^ for OTB-GSH and 3575 L mol^−1^ cm^−1^ for OTB-NAC.

To analyze human urine samples for the occurrence of these potential OTA metabolites, we chose the application of a stable isotope dilution analysis to compensate for analyte losses during cleanup as well as matrix effects during HPLC-MS/MS analysis by the addition of stable isotope-labeled internal standards to the urine samples prior to the cleanup procedure. To that end, small quantities of the stable isotope-labeled compounds *d*_*5*_-OTB-GSH as well as *d*_*5*_-OTB-NAC were synthesized following the same protocol reported for OTB-GSH and OTB-NAC but starting from *d*_*5*_-OTA. Identities of these substances were proven by comparison of the HRMS mass spectra and HPLC retention times with those from the non-labeled references. Concentrations of the stock solution of the stable isotope-labeled standards were determined using the above-reported extinction coefficients of OTB-GSH and OTB-NAC. The synthesized reference compounds were used for the development of a HPLC-MS/MS method. During optimization of the MS parameters, a relative decrease of the ionization efficiency by a factor of ten for the OTA conjugates OTB-NAC and OTB-GSH compared to OTA was observed.

**Fig. 2:**
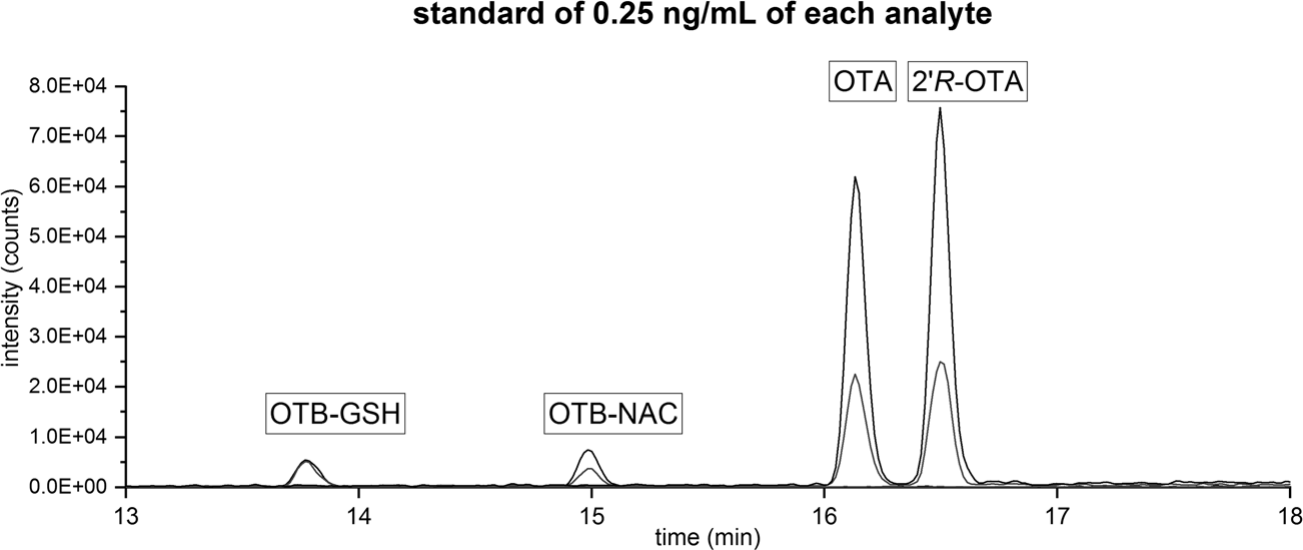
Extracted ion HPLC-MS/MS chromatogram of each 0.25 ng mL^−1^ OTB-GSH, OTB-NAC, OTA, and 2′*R*-OTA from the calibration: black lines: quantifier; gray line: qualifier 1 (Table 1)

Besides OTA, also the diastereomer 2′*R*-ochratoxin A (2′*R*-OTA), which is formed during coffee roasting and indicates coffee consumption, was included in the method development as the analysis was performed within the framework of a study focusing on this compound (Sueck et al. 2018a). A HPLC-MS/MS chromatogram of a standard mixture containing all four analytes in a concentration of 0.25 ng mL^−1^ is shown in Fig. 2.

For OTA, 2′*R*-OTA, OTB-GSH, and OTB-NAC analysis, human spot urine samples from 18 apparently healthy individuals (10 females, 8 males; mean age 28.1 ± 5.3 years, mean BMI 23.4 ± 2.9 kg m^−2^) were analyzed. Therefore, each urine sample was spiked with the internal standards *d*_*5*_-OTA, *d*_*5*_-2′*R*-OTA, *d*_*5*_-OTB-GSH, and *d*_*5*_-OTB-NAC, concentrated by a factor of 50 using SPE and analyzed by HPLC-MS/MS.

**Fig. 3:**
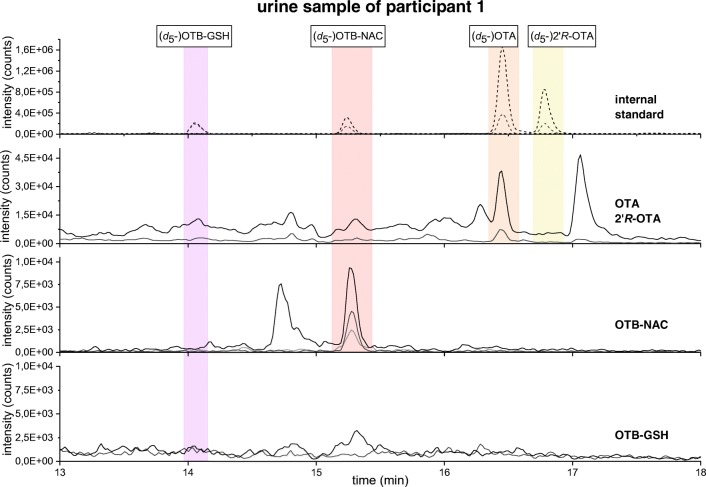
Extracted ion HPLC-MS/MS chromatogram of the four internal standards (top), OTA and 2′*R*-OTA, OTB-NAC, and OTB-GSH traces (bottom) in the urine sample of participant 1: black: SRM transitions of the quantifier ions; gray: SRM transitions of the qualifier ions (Table 1)

OTB-NAC could be identified in 11 out of 18 human urine samples (3 male and 8 female) and was quantified in 5 out of 18 samples (2 male and 3 female) in concentrations between 0.039 and 0.176 ng mg^−1^ creatinine (Table 4). In Fig. 3, the chromatogram of a urine sample from participant 1 containing 0.176 ng mg^−1^ creatinine OTB-NAC is shown.

**Table 4:** OTA and OTB-NAC concentrations in human urine samples. SEM represents the standard deviation of two replicates (*single analysis of the human urine; n.d, not detectable; < LOQ, under limit of quantitation). Mean was determined only from quantifiable concentrations

Participant (sex)	OTA (ng mg^−1^ creatinine)	± SEM (ng mg^−1^ creatinine)	OTB-NAC (ng mg^−1^ creatinine)	±SEM (ng mg^−1^ creatinine)
1 (female)	0.105	0.007	0.176	0.060
2 (female)	0.081	*	< LOQ	
3 (female)	0.088	*	< LOQ	
4 (male)	0.090	*	< LOQ	
5 (male)	0.132	0.032	0.159	0.009
6 (male)	0.097	0.001	0.039	0.003
7 (female)	0.145	*	< LOQ	
8 (female)	0.404	*	< LOQ	
9 (female)	0.067	*	< LOQ	
10 (female)	0.068	0.005	0.055	0.008
11 (female)	0.098	0.006	0.023	0.000
12 (female)	< LOQ		n.d.	
13 (male)	0.113	*	n.d.	
14 (male)	0.158	*	n.d.	
15 (female)	< LOQ		n.d.	
16 (male)	< LOQ		n.d.	
17 (male)	n.d.		n.d.	
18 (male)	n.d.		n.d.	
Mean	0.127	0.084	0.092	0.008

The potential OTA metabolite OTB-GSH could not be detected in any sample. OTA was identified in 16 out of 18 urine samples and could be quantified in 13 out of 18 samples in concentrations between 0.067–0.404 ng mg^−1^ creatinine (Table 4). The diastereomer 2′*R*-OTA was not found in any urine sample.

Comparison of the amounts of OTB-NAC and OTA found in the urine samples revealed that the parent compound OTA and its metabolite OTB-NAC are excreted in comparable concentration ranges. Thus, the formation of OTB-NAC might help to explain the “metabolite gap” described by Studer-Rohr et al. (2000) who observed that when radiolabeled OTA was ingested by one human volunteer, only 42–54% of the radioactivity found in the chloroform extract of the urine could be attributed to unchanged OTA.

Among the small cohort, OTB-NAC was equally distributed between male and female participants, but due to the limited data, so far, no conclusion regarding a possible similarity to the observed difference between male and female rats can be drawn (Tozlovanu et al. 2012).

The detection of OTB-NAC in human urine leads to the conclusion that the metabolite OTB-GSH can be formed in significant quantities in the human body and further be metabolized to OTB-NAC (Nelson 1992; Ringot et al. 2006). Whether the conjugation reaction between OTA and GSH requires in a first step the formation of a reactive quinone or if it is directly catalyzed by GST is so far not known. As can be seen from Table 4, no correlation between urinary OTA concentrations and OTB-NAC formation was observed. Participants 7, 8, 13, and 14 had relatively high OTA levels above 0.1 ng mg-1 creatinine; however, OTB-NAC was not detectable in these samples. This clearly indicates that other factors might influence the metabolic pathway of OTA in the human body.

In summary, the two potential OTA metabolites OTB-NAC and OTB-GSH have successfully been synthesized and their structures characterized by NMR spectroscopy. Moreover, their isotope-labeled analogues *d*_*5*_-OTB-GSH and *d*_*5*_-OTB-NAC were synthesized to provide stable isotope-labeled standards for sample cleanup and HPLC-MS/MS analysis.

Following method development, the OTA metabolite OTB-NAC was for the first time detected and quantified in human urine. OTB-NAC concentrations of up to 0.176 ng mg^−1^ creatinine were comparable to those found for OTA (0.067–0.404 ng mg^−1^ creatinine) indicating that the conjugation of OTA with GSH and subsequent conversion to OTB-NAC is a relevant metabolic pathway for OTA excretion.

## Electronic supplementary material

ESM 1 (PDF 641 kb)
