# Supplementary material for: Identification of ochratoxin-N-acetyl-L-cysteine as a new ochratoxin A metabolite and potential biomarker in human urine
Source: Mycotoxin Res. 2019 May 10;36(1):1–10. doi: 10.1007/s12550-019-00360-0 (PMC6971137; doi:10.1007/s12550-019-00360-0)
Supplement: Supplementary file 1 — (PDF 641 kb) [file 12550_2019_360_MOESM1_ESM.pdf]

Supplementary Information

Identification of OTB-*N*-acetyl-L-cysteine as a new ochratoxin A metabolite and potential biomarker in human urine

Franziska Sueck, Jonas Specht, Benedikt Cramer and Hans-Ulrich Humpf*

Institute of Food Chemistry, Westfälische Wilhelms-Universität Münster, Corrensstr. 45, 48149 Münster, Germany

(*corresponding author: humpf@wwu.de; +49 251 83 33391)

HRMS data

The exact mass and the most intensive fragments of each analyte is given in Table S1:

Table S1: Molecular formula, exact masses and the most intensive fragments of the synthesised analytes from HRMS using HCD 35% relative energy.

**analyte**	**mode**	**molecular formula**	**exact mass (*m*/*z*)**	**fragments (*m*/*z*)**
OTB-GSH	positive	$[C_{30}H_{34}N_4O_{12}S+H]^+$	found 675.1967 (calcd. 675.1967)	600.1639, 447.0851, 353.0799, 296.0585
OTB-NAC	positive	$[C_{25}H_{26}N_2O_9S+H]^+$	found 531.1422 (calcd. 531.1432)	384.0746, 366.0640, 255.013, 237.0213
d_5-OTB-GSH	positive	$[C_{30}H_{29}D_5N_4O_{12}S+H]^+$	found 680.2279 (calcd. 680.2281)	605.1958, 353.0801, 296.0587, 278.0481
d_5-OTB-NAC	positive	$[C_{25}H_{21}D_5N_2O_9S+H]^+$	found 536.1735 (calcd. 536.1746)	384.0742, 366.0636, 255.0318, 237.0213
OTHQ-GSH	negative	$[C_{30}H_{34}N_4O_{13}S-H]^-$	found 689.1762 (calcd. 689.1770)	-
OTHQ-NAC	negative	$[C_{25}H_{26}N_2O_{10}S-H]^-$	found 545.1238 (calcd. 545.1235)	-

UV data

The UV absorption maxima of all four analytes, (d_5-)OTB-GSH and (d_5-)OTB-NAC is 331 nm. This observation agrees with the results from Tozlovanu et al. 2012.

NMR data

OTB-NAC

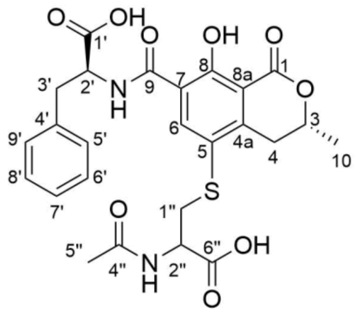

Figure S1: Chemical structure of OTB-NAC with atom labelling.

^{1}H NMR (600 MHz, $CD_3OD/D_2O/DCOOD$ (75/25/0.1; *v*/*v*/*v*)) δ 8.40 (s, 1H, H-6), 7.31 – 7.22 (m, 5H, H-5'-9'), 4.93 (dd, *J* = 7.0, 5.3 Hz, 1H, H-2'), 4.39 (dd, *J* = 8.5, 4.3 Hz, 1H, H-2''), 3.52 (dd, *J* = 17.2, 3.2 Hz, 1H, H-4.2), 3.38 (dd, *J* = 14.1, 4.4 Hz, 1H, H-1''.2), 3.34 (m, 1H, H-3'.2), 3.20 (dd, *J* = 13.8, 7.2 Hz, 1H, H-3'.1), 3.14 (dd, *J* = 14.1, 8.5 Hz, 1H, H-1''.1), 2.92 (dd, *J* = 17.2, 11.7 Hz, 1H, H-4.1), 1.96 (s, 3H H-5''), 1.55 (d, *J* = 6.3 Hz, 3H, H-10).
δ 4.80 (H-3) overlapping with water signal, identified by HMBC.

^{13}C NMR (151 MHz, $CD_3OD/D_2O/DCOOD$ (75/25/0.1; *v*/*v*/*v*)) δ 174.7 (C-1'), 173.6 (C-4''), 171.6 (C-1), 165.1 (C-9), 164.9 (C-6''), 161.2 (C-8), 149.0 (C-4a), 144.3 (C-6), 137.5 (C-4'), 130.4 (C-5'/9' or C-6'/8'), 129.5 (C-5'/9' or C-6'/8'), 128.1 (C-7'), 124.1 (C-5), 120.5 (C-7), 111.2 (C-8a), 77.8 (C-3), 55.6 (C-2'), 53.5 (C-2''), 38.2 (C-3'), 37.8 (C-1''), 34.0 (C-4), 22.5 (C-28), 20.8 (C-10).

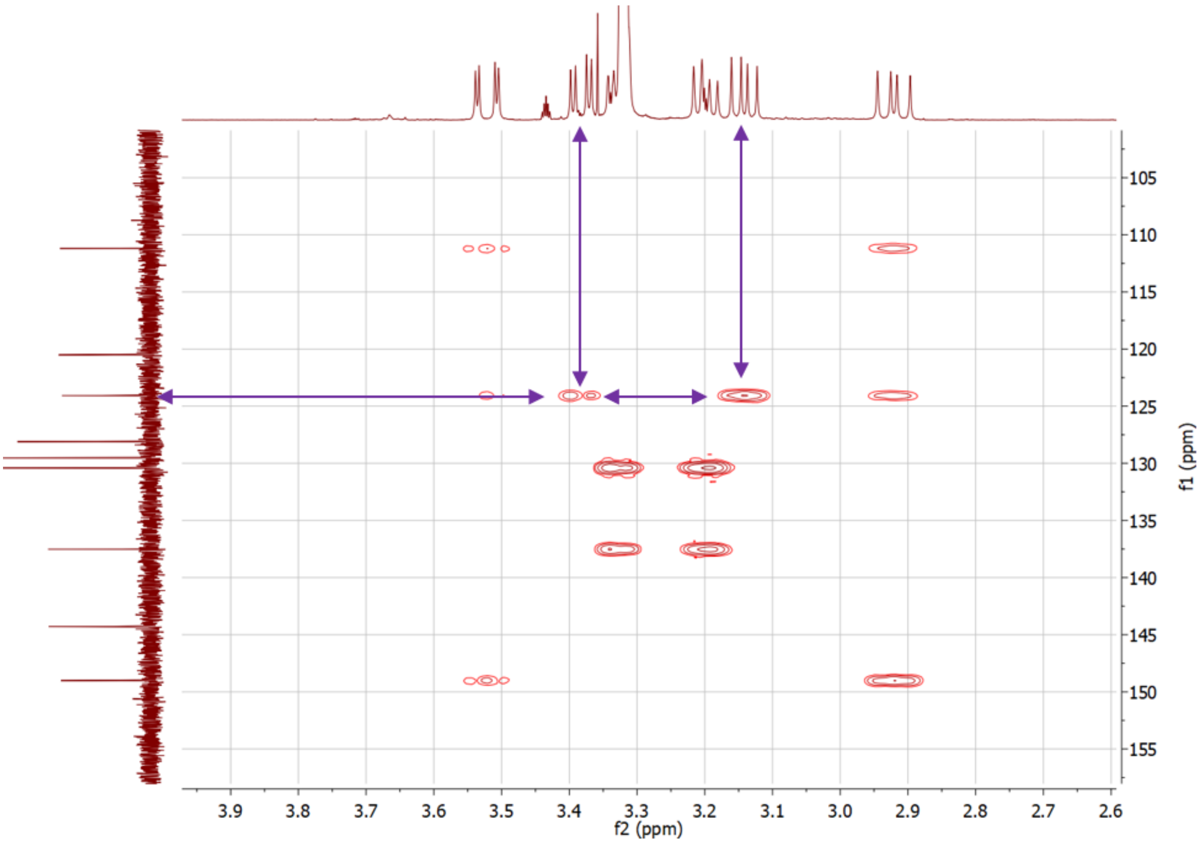

Figure S2: ^{1}H,^{13}C-HMBC-spectra of OTB-NAC. The $^{3}J_{C,H}$ coupling of H-1''.1 (δ 3.14) and H-1''.2 (δ 3.38) with the C-5 (δ 124.1) are marked with arrows.

OTB-GSH

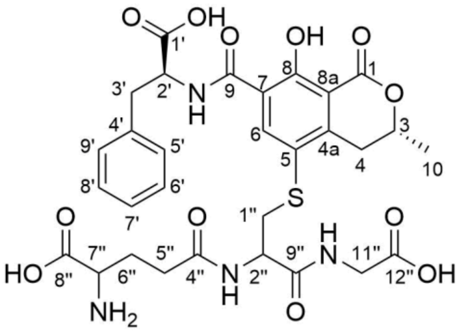

Figure S3: Chemical structure of OTB-GSH with atom labelling.

^{1}H NMR (600 MHz, $CD_3OD/D_2O/DCOOD$ (75/25/0.1; *v*/*v*/*v*)) δ 8.35 (s, 1H, H-6), 7.31 – 7.18 (m, 5H, H-5’-9’), 4.27 (dd, *J* = 9.8, 4.5 Hz, 1H, H-2’’), 3.81 (d, *J* = 2.5 Hz, 2H, H-11’’), 3.69 (t, *J* = 6.3 Hz, 1H, H-7’’), 3.55 (dd, *J* = 17.2, 3.2 Hz, 1H, H-4.2), 3.34 (dd, *J* = 3.1, 1.3 Hz, 1H, H-1’’.2), 3.18 (dd, *J* = 14.0, 7.2 Hz, 1H, H-3’.1), 3.03 (dd, *J* = 14.1, 9.9 Hz, 1H, H-1’’.1), 2.98 (dd, *J* = 17.2, 11.8 Hz, 1H, H-4.1), 2.55 (t, *J* = 7.2 Hz, 2H, H-5’’), 2.23 – 2.08 (m, 2H, H-6’’), 1.56 (d, *J* = 6.3 Hz, 3H, H-10).

δ 4.85 (H-2’) and 4.79 (H-3) overlapping with water signal; δ 3.32 (H-1’’.2) and 3,31 (H-3’.2) overlapping with methanol signal but identified by HMBC.

^{13}C NMR (151 MHz, $CD_3OD/D_2O/DCOOD$ (75/25/0.1; *v*/*v*/*v*)) δ 175.9* (C-1’), 175.3 (C-4’’), 174.1* (C-8’’), 173.9* (C-12’’), 172.7 (C-9’’), 171.6 (C-1), 165.3 (C-9), 161.4* (C-8), 149.4 (C-8a), 144.8 (C-6), 138.0 (C-4’), 130.5 (C-15’/9’), 129.5 (C-6’/8’), 127.9 (C-7’), 123.2* (C-5), 120.5 (C-7), 111.3 (C-4a), 77.8 (C-3), 56.5 (C-2’), 55.3* (C-7’’), 53.8 (C-2’’), 42.5 (C-11’’), 38.4 (C-3’), 37.4 (C-1’’), 33.9 (C-4), 32.7 (C-5’’), 27.5 (C-6’’), 20.9 (C-10). * identified by HMBC

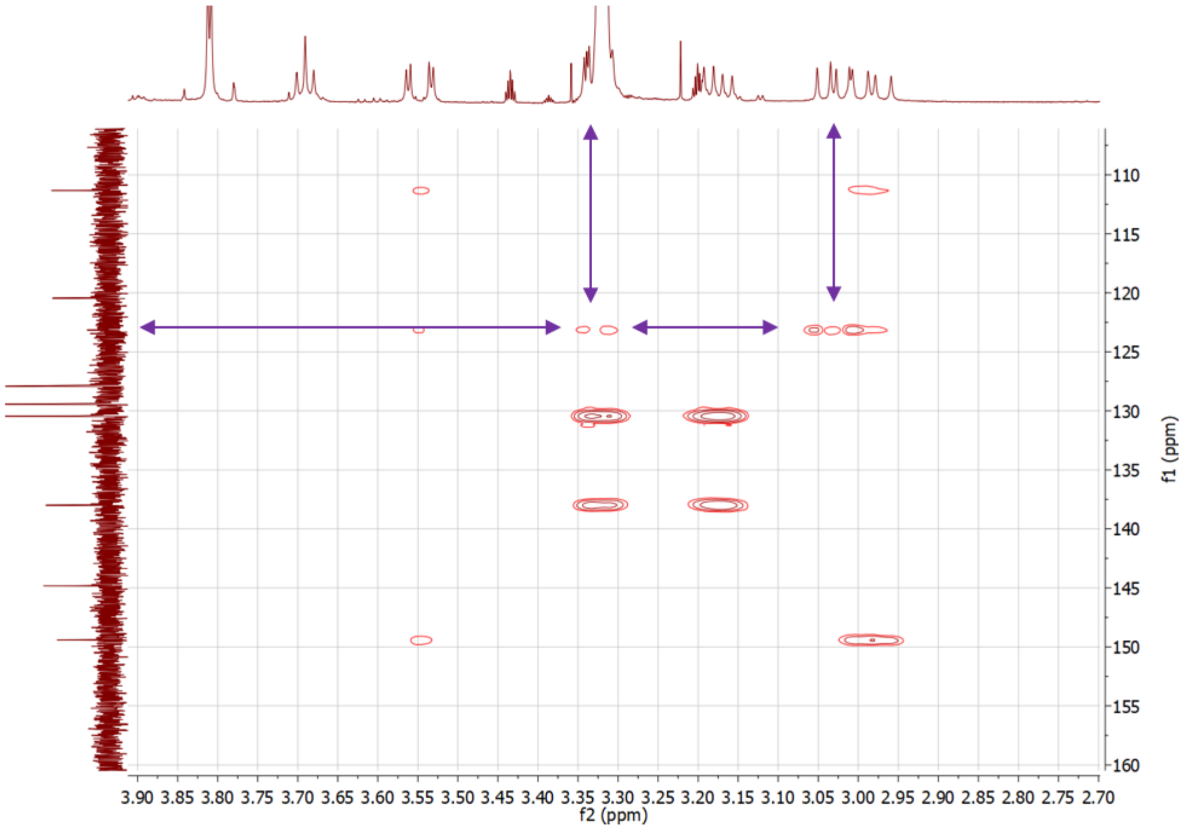

Figure S4: ^{1}H,^{13}C-HMBC-spectra of OTB-GSH. The $^{3}J_{C,H}$ coupling of H-1''.1 (δ 3.03) and H-1''.2 (δ 3.32) with the C-5 (δ 123.2) are marked with arrows.
